# Neuromuscular electrical stimulation for obstructive sleep apnoea: comparing adherence to active and sham therapy

**DOI:** 10.1183/23120541.00474-2023

**Published:** 2023-12-27

**Authors:** Alexandre R. Abreu, Darko Stefanovski, Susheel P. Patil, Trishul Siddharthan, Alejandro Chediak, Douglas M. Wallace, Naresh M. Punjabi

**Affiliations:** 1 Division of Pulmonary, Critical Care, and Sleep Medicine, University of Miami, Miami, FL, USA; 2 Department of Clinical Studies–New Bolton Center, University of Pennsylvania, Philadelphia, PA, USA; 3 Division of Pulmonary, Critical Care, and Sleep Medicine, Case Western Reserve University, Cleveland, OH, USA; 4 Department of Neurology, University of Miami, Miami, FL, USA

## Abstract

**Background:**

Mild obstructive sleep apnoea (OSA) is a common disorder associated with daytime sleepiness and impaired quality of life. Given that adherence to positive airway pressure (PAP) therapy in OSA is suboptimal, alternative strategies are needed particularly for patients with mild OSA. Daytime neuromuscular electrical stimulation (NMES) of the tongue is a new therapeutic modality for mild OSA. The objective of this study was to determine if patients with mild OSA adhere to daytime NMES.

**Methods:**

A randomised, sham-controlled, double-masked controlled trial was conducted in 40 patients with mild OSA who received either high-intensity (active) or low-intensity (sham) NMES for 6 weeks. The primary end-point was adherence to therapy. Exploratory outcomes included the respiratory event index (REI) and the Epworth Sleepiness Scale (ESS) score.

**Results:**

More than 90% of participants in each arm were adherent to NMES. Exploratory analyses revealed a 32.7% (95% CI 15.5–49.9%) drop in the REI with active NMES, with no significant change in the REI with sham NMES. Improvements were larger in the supine than non-supine REI. Both the apnoea index and hypopnoea index improved with active NMES. Finally, the ESS score improved with active but not with sham NMES.

**Conclusions:**

Daytime NMES was well accepted, with a majority using it for the recommended period. NMES of the tongue use was associated with improvements in OSA severity and daytime sleepiness. Additional research is needed to define its role in the treatment armamentarium across the spectrum of OSA severity and in patients who are intolerant to PAP therapy.

## Tweetable abstract

**In this randomised controlled trial, patients with mild obstructive sleep apnoea (OSA) were noted to adhere to daytime neuromuscular electrical stimulation, which led to improvements in OSA severity indexes and daytime sleepiness** https://bit.ly/3s4gKx2

## Introduction

Obstructive sleep apnoea (OSA) is a common disorder characterised by repetitive collapse of the upper airway during sleep [1]. Of the available modalities, positive airway pressure (PAP) therapy is considered first-line therapy, particularly for moderate-to-severe OSA. PAP therapy in OSA is associated with improvements in daytime sleepiness, quality of life and blood pressure [2]. While the effects of PAP therapy on cardiovascular end-points remain a topic of significant controversy, data from randomised clinical trials suggest that it may reduce the risk of cardiovascular events if nightly use is at least 4 h [3]. Despite numerous technical advances such as the availability of different mask interfaces, quieter airflow generators and greater portability, adherence to PAP therapy remains a major challenge [4, 5]. Non-adherence is as high as 50% within the first week of starting PAP therapy [6]. Studies assessing long-term use of PAP therapy show that adherence is in the range of 30–60% [4].

Given that many patients abandon PAP therapy, considerable effort has been made in identifying alternative treatment strategies. A recent addition to the therapeutic armamentarium is the use of daytime neuromuscular electrical stimulation (NMES) of the tongue. NMES has been used as a strength training technique and as a rehabilitation tool in the clinical arena to increase muscle strength and endurance [7]. Improving endurance of the glossal muscles with NMES could decrease upper airway collapsibility during sleep. In fact, a proof-of-concept study showed that a 20-min daytime NMES session for 6 weeks led to a decrease in bed-partner-reported intensity of snoring [8]. In a subsequent larger, but uncontrolled study, daytime NMES decreased snoring intensity [9] and improved the oxygen desaturation index in mild OSA [10, 11]. The ease of a daytime approach for treating OSA has the advantage of improving patient adherence. In light of the tenet that poor adherence is associated with worse outcomes [12], a fundamental question regarding NMES is whether patients with OSA will use it consistently. Before engaging in large clinical trials on NMES in OSA, a rigorous examination of adherence is needed. Therefore, the primary objective of this study was to assess adherence to daytime NMES in patients with mild OSA. An additional objective was to develop a sham intervention that would mimic active NMES in terms of treatment duration and frequency, but without treatment-specific effects. Availability of a sham device would permit a careful appraisal of whether NMES is effective in treating OSA across the continuum of disease severity. A randomised clinical trial was designed to determine adherence to daytime NMES (active and sham) in mild OSA. In addition to assessing adherence, exploratory outcomes included improvements in disease severity and daytime sleepiness.

## Methods

### Study population

A 6-week, randomised, sham-controlled, double-masked trial of daytime NMES with the eXciteOSA device (Signifier Medical Technologies, London, UK) was conducted in patients with newly diagnosed mild OSA. Participants were recruited from patients referred to a university-based sleep centre (Division of Pulmonary, Critical Care, and Sleep Medicine, University of Miami, Miami, FL, USA) for clinical evaluation. Exclusion criteria included age <18 years, pregnancy, oropharyngeal abnormalities and presence of oral implants, metal prostheses, dental braces or soft tissue/bony ulcerations. Patients previously treated for OSA with an oral appliance, PAP device or oropharyngeal surgery were ineligible. Additional exclusionary criteria included lack of a smartphone, cognitive impairments or prevalent medical conditions, including heart failure, coronary artery disease, uncontrolled hypertension, chronic lung disease, chronic fatigue syndrome, fibromyalgia, use of illicit drugs and a central apnoea index ≥5 events·h^−1^. Eligibility also required a new diagnosis of mild OSA based on either an in-lab sleep study or a home sleep apnoea test. However, enrolment did not require presence of any clinical symptoms. Mild OSA was defined as a respiratory event index (REI) in the range of 5.0–14.9 events·h^−1^ on the in-lab study or home sleep apnoea test. Eligible patients meeting these eligibility criteria had to then complete three nights of home testing with a Nox A1 device (Nox Medical, Reykjavik, Iceland) to confirm mild OSA. The study was approved by the Institutional Review Board of the Miller School of Medicine, University of Miami (approval: 20210068) and all participants provided written consent. The trial was registered at ClinicalTrials.gov prior to initiation (NCT04974515).

### Home sleep apnoea test

Although qualified participants had a clinical test demonstrating mild OSA, enrolment required confirmation of mild OSA based on multi-night home testing given the night-to-night variability in the REI. The assessment for mild OSA was conducted at home using a self-applied recorder (Nox A1). The montage included a nasal cannula, chest and abdominal respiratory impedance bands, and pulse oximetry for oxyhaemoglobin saturation (*S*_pO_2__). Data collected from all three nights were downloaded for manual scoring. Apnoeas were identified if there was an absence or near absence of airflow for at least 10 s. Hypopnoeas were defined as a reduction in airflow of ≥30% for ≥10 s with a decrease in oxygen saturation of ≥3%. At least 3 h of scorable data on the pulse oximetry and the chest and abdominal belts were required for any recording to be considered successful. At least two successful nights were mandatory for enrolment. The REI was calculated as the number of apnoeas and hypopnoeas per hour of total recording period. A median REI from multi-night testing between 5.0 and 14.9 events·h^−1^ was required for randomisation.

### Study intervention and outcomes

Participants were randomised 1:1 to active or sham NMES using a simple allocation randomisation. The allocation sequence was generated using a computerised algorithm with a random block size and concealed until randomisation. A physician not affiliated with the local study team (S.P. Patil) assigned participants to a study arm. Both arms were provided the eXciteOSA device which has four stimulation electrodes, with two located above and two below the tongue. Participants and members of the research team were masked to treatment assignment. The device provides NMES on an intensity scale of 1–15 that consists of a series of electrical pulses with rest periods in between. In both arms, participants could increase the setting and instructions were provided to advance to the highest level as tolerated based on the prior session. If the stimulation at any setting was excessive, participants could adjust the level down even during a session. At the highest setting of 15, the maximum voltage levels for the active (high-intensity) and sham (low-intensity) devices were 32.8 and 6.6 V, respectively (supplementary material). Participants had to use the device for a continuous 20-min period at any time during wakefulness for 6 weeks. Data on device use were recorded nightly and transmitted to a central database through a smartphone. At the end of 6 weeks, the three nights of Nox A1 home recordings were repeated. Self-reported data on the Epworth Sleepiness Scale (ESS) [13] were collected at baseline and at 6 weeks. The primary outcome was the proportion of participants who were adherent to therapy. Adherence was defined by the number of days the device was used out of 42 required days. Exploratory outcomes included the REI (overall, supine and non-supine) and the ESS score. Adherence to therapy was not financially incentivised and patient engagement followed the usual clinical practice routine.

### Statistical analyses

Given that the objective was to assess adherence, sample size calculations were made based on estimating the primary outcome (proportion adherent) with a given level of precision. A sample size of 20 in each arm was needed assuming that the proportion adherent would be ∼0.85 and the 95% probability that the confidence interval (CI) was ≤0.24. Analyses of the primary outcome were conducted using the intention-to-treat principle. Descriptive analyses of continuous variables included computation of mean with standard deviation and median with interquartile range. Tests of normal distribution (Shapiro–Wilk) were performed to assess skewness of continuous variables. Characteristics were compared by assigned arm using ANOVA for continuous variables and Chi-squared for categorical variables. A patient was considered adherent for each day if the device was used for 20 min. The proportion of participants who were adherent to therapy over the 42 days in each arm was determined. In exploratory analyses, change in the REI (overall, supine and non-supine) and the ESS score was examined. For these analyses, generalised mixed effects linear regression models were used with the arm as the fixed effect and the patient as a random effect. Because the distribution of the REI is right skewed, the robust estimation of the standard errors was used to address any departures from normality of residuals. *Post hoc* model adjusted marginal means were used for inferences regarding treatment effects. In addition, a mixed effects Poisson regression was used to determine the relative change in the REI. The least significant difference method was used to adjust the significance levels for multiple comparisons. Because the REI and oxygen desaturation index were highly correlated (r=0.99), only data on change in the REI are presented. Finally, a Bayesian approach to the mixed linear model was used to characterise the change in ESS scores. A two-sided p-value <0.05 was used to indicate statistical significance. All analyses were conducted using Stata version 17.0 (StataCorp, College Station, TX, USA).

## Results

**FIGURE 1:**
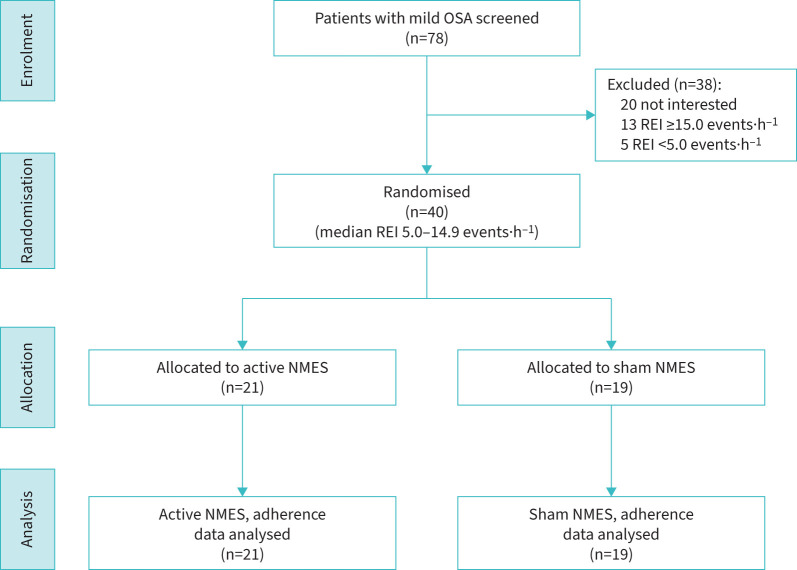
Flow diagram showing the participants screened, randomised and followed during the trial. OSA: obstructive sleep apnoea; REI: respiratory event index; NMES: neuromuscular electrical stimulation.

A total of 78 participants with mild OSA based on an in-lab study (n=37) or home sleep apnoea test (n=41) were screened (figure 1). Of these, 40 participants with mild OSA were randomised (21 to the active arm and 19 to the sham arm). The mean±sd age and body mass index (BMI) of the sample was 52.3±12.9 years and 28.3±4.8 kg·m^−2^, respectively. Self-identified race, age, sex, race, ethnicity and BMI were comparable between the two arms (table 1). OSA severity, as assessed by the REI, was also similar between the two arms. All enrolled patients were counselled that the required duration of NMES treatment would be 42 days.

**TABLE 1:** Characteristics of the study sample by arm

	**Active NMES (n=21)**	**Sham NMES (n=19)**
**Age (years)**	50.5±14.8	54.4±10.6
**Male**	11 (47.6)	12 (36.8)
**Race**		
White	19 (90.5)	17 (89.5)
Black	2 (9.5)	2 (10.5)
**Hispanic**	13 (61.9)	11 (57.9)
**BMI (kg·m^−2^)**	28.4±5.2	28.9±4.5
**BMI categories**		
<25 kg·m^−2^	7 (33.3)	4 (21.5)
25.0–29.9 kg·m^−2^	7 (33.3)	9 (47.4)
≥30 kg·m^−2^	7 (33.3)	6 (31.6)
**Respiratory event index (events·h^−1^)**	12.8±6.5	12.2±3.9
**Epworth Sleepiness Scale score**	8.2±5.8	10.3±5.3

Data are presented as mean±sd or n (%). NMES: neuromuscular electrical stimulation; BMI: body mass index.

**FIGURE 2:**
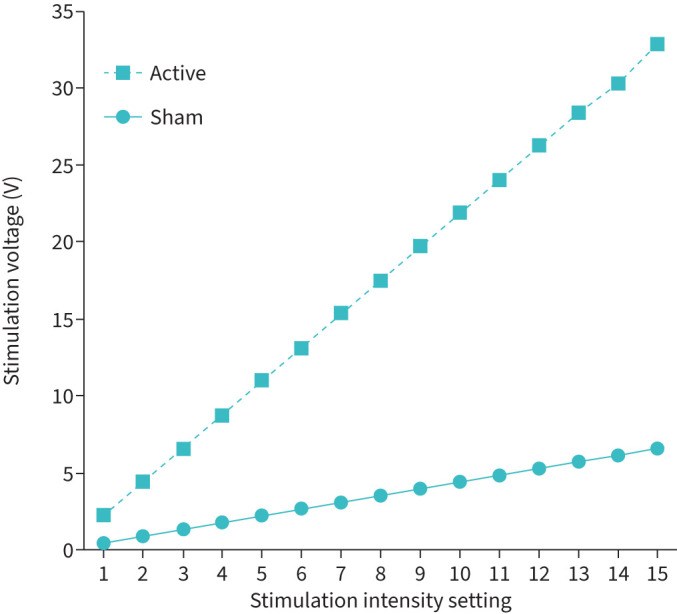
Stimulation intensity and voltage delivered with neuromuscular electrical stimulation.

Analysis of the primary end-point of adherence based on daily use was available for all 40 participants (table 2). In the active and sham arms, the NMES device was used for an average of 38.4 (95% CI 35.0–41.7) and 37.9 (95% CI 34.0–41.9) days, respectively (p=0.86 for the difference). Duration of use per day was also comparable between the two arms (p=0.62). Of all the days the device was used in the active (n=808) and sham (n=721) arms, the duration of use was <20 min on only 7 (0.9%) and 2 (0.3%) days, respectively (p=0.13). Table 2 also shows the distribution of adherent participants in each arm based on various definitions. Approximately 90% of the participants in each arm used the device for ≥70% of the required days, with no difference between the two arms (p=0.73). However, the stimulation setting used was different, with the active arm using a lower level than the sham arm (9.8 *versus* 13.4; p<0.001). The association between the stimulation setting and voltage delivered is shown in figure 2.

**TABLE 2:** Adherence to neuromuscular electrical stimulation (NMES)

	**Active NMES (n=21)**	**Sham NMES (n=19)**	**p-value**
**Days used**	38.4±7.4	38.0±8.3	0.86
**Duration of use per day (min)**	20.0±0.01	20.0±0.01	0.62
**Percentage of days device used**			0.73
100% (42 days)	13 (61.9)	13 (68.4)	
90–99% (38–41 days)	4 (19.1)	1 (5.3)	
80–89% (33–37 days)	1 (4.7)	2 (10.5)	
70–79% (29–32 days)	1 (4.7)	1 (5.3)	
<70% (<29 days)	2 (9.5)	2 (10.5)	
**Stimulation intensity setting**	9.8±3.2	13.4±2.5	<0.001

Data are presented as mean±sd or n (%), unless otherwise stated.

**FIGURE 3:**
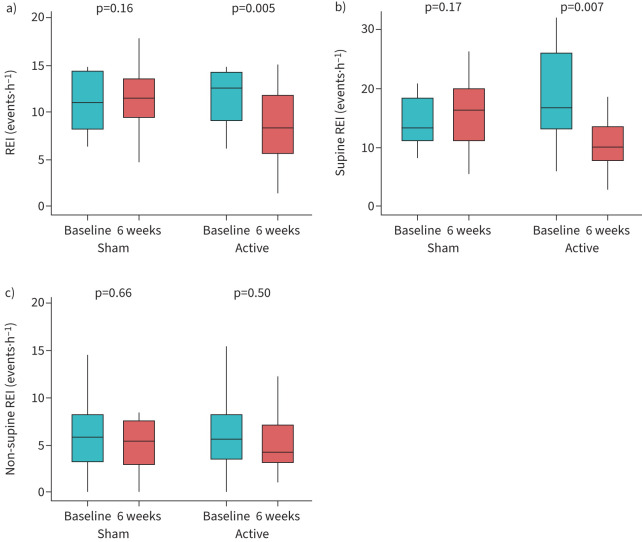
Distribution of the a) overall, b) supine and c) non-supine respiratory event index (REI) at baseline and after 6 weeks of neuromuscular electrical stimulation.

Exploratory analyses were conducted to examine whether NMES was associated with changes in OSA severity. Figure 3 shows the overall, supine and non-supine median REI from the baseline and the 6-week home studies. In the active arm, the REI improved from 12.9 to 9.6 events·h^−1^ (p<0.001) (table 3), representing a relative reduction of 32.7% (95% CI 15.5–49.9%) and an absolute reduction of 3.3 (95% CI 1.7–5.0) events·h^−1^. In the sham arm, the REI remain unchanged (11.5 *versus* 12.6 events·h^−1^; p=0.25). The decrease in the REI in the active arm was greatest in the supine position from 19.9 (95% CI 16.1–23.6) to 14.5 (95% CI 9.7–19.3) events·h^−1^, representing a relative reduction of 34.7% (95% CI 6.6–62.8%) and an absolute reduction of 5.3 (95% CI 1.1–9.6) events·h^−1^. While the non-supine REI also improved slightly in the active arm, the difference was smaller than the change in the supine REI but statistically significant (7.4 *versus* 5.1 events·h^−1^; p=0.02). The supine and non-supine REI in the sham arm remain unchanged after 6 weeks.

**TABLE 3:** Measures of obstructive sleep apnoea severity at baseline and at 6 weeks in the active and sham neuromuscular electrical stimulation (NMES) arms

	**Active NMES (n=21)**				**Sham NMES (n=19)**			
	**Baseline**	**6 weeks**	**Δ_Active_**	**p-value**	**Baseline**	**6 weeks**	**Δ_Sham_**	**p-value**
**REI (events·h^−1^)**								
Overall	12.9±1.0	9.6±1.2	−3.3±0.9	0.001	11.5±0.7	12.6±1.2	+1.1±1.0	0.253
Supine	19.9±1.9	14.5±2.5	−5.3±2.2	0.013	14.9±1.0	16.3±1.5	+1.3±1.4	0.334
Non-supine	7.4±1.2	5.1±1.0	−2.3±1.0	0.020	6.9±1.1	7.6±1.6	+0.7±0.8	0.380
**Apnoea index (events·h^−1^)**								
Overall	2.0±0.3	1.2±0.3	−0.7±0.2	0.002	2.3±0.3	1.8±0.4	−0.5±0.3	0.088
Supine	4.3±1.3	2.5±1.2	−1.9±0.8	0.156	3.2±0.7	2.9±1.0	−0.4±0.8	0.652
Non-supine	0.6±0.1	0.8±0.3	+0.2±0.3	0.468	1.3±0.3	0.9±0.3	−0.4±0.1	0.002
**Hypopnoea index (events·h^−1^)**								
Overall	11.0±0.9	8.3±1.0	−2.7±0.8	0.002	9.2±0.6	10.9±1.0	+1.6±0.9	0.060
Supine	15.6±1.2	12.2±2.0	−3.4±1.7	0.050	11.8±0.8	13.5±1.2	+1.7±1.0	0.103
Non-supine	6.8±1.1	4.3±0.9	−2.5±0.8	0.001	5.6±0.9	6.6±1.5	+1.0±0.8	0.204

Data are presented as marginal mean±se adjusted for age (continuous (years)), sex, race, body mass index (continuous (kg·m^−2^)) and duration in supine position (continuous (min)). REI: respiratory event index. p-values are derived for comparing the baseline and 6-week values in each group (active and sham) from the mixed linear regression model.

Active NMES was also associated with an improvement in the overall apnoea index (2.0 *versus* 1.2 events·h^−1^; p=0.002). The change in apnoea index in the active arm in the supine position (4.3 *versus* 2.5 events·h^−1^; p=0.16) and non-supine position (0.6 *versus* 0.8 events·h^−1^; p=0.47) did not reach statistical significance. While no change in the overall or the supine apnoea index were noted in the sham arm, a small and statistically significant improvement in the non-supine apnoea index (1.3 *versus* 0.9 events·h^−1^; p=0.002) was noted. The overall hypopnoea index decreased with active NMES (11.0 *versus* 8.3 events·h^−1^; p=0.002), but increased with sham NMES (9.2 *versus* 10.9 events·h^−1^; p=0.06). Both the supine and non-supine hypopnoea index also improved with active, but not sham, NMES. Finally, the ESS scores decreased from 8.3 to 6.3 (ΔESS: −1.8 (95% CI −3.8–0.2)) in the active arm, albeit with marginal statistical significance (p=0.074). There was no change in the ESS scores in the sham arm (10.3 *versus* 9.2; p=0.44). Given the limited sample size and the marginal statistical significance for changes in the ESS, sensitivity analyses were conducted using a Bayesian mixed effects model (see supplementary material). The Bayesian approach confirmed that participants in the active arm reported improvements in daytime sleepiness (ΔESS: −1.8 (95% credibility interval −0.8– −2.8)), whereas no significant change was noted in the sham arm (ΔESS: −0.9 (95% credibility interval −1.9–0.2)).

## Discussion

The results of this randomised, double-masked, sham-controlled study demonstrate that NMES for 20 min per day for 6 weeks in mild OSA was well accepted, with a majority of participants using NMES for ≥70% of the recommended time. NMES was also associated with ∼33% improvement in OSA severity after accounting for patient-related factors such as age, sex, race, BMI and time in the supine position. Improvements in OSA severity were most notable in the supine rather than the non-supine position. In contrast, OSA severity remained unchanged in participants who received sham NMES. Finally, participants in the active arm experienced improvements in daytime sleepiness, whereas no such change was noted in the sham arm.

While PAP therapy is first-line therapy for moderate-to-severe OSA, mild symptomatic disease can be effectively treated with PAP, mandibular advancement, positional therapy and weight loss. The use of NMES is a recent addition to the complement of treatment modalities for mild OSA. Previous work on PAP therapy in mild OSA shows that treatment is associated with symptomatic benefit [14] and thus argues against the misconception that patients are not likely to experience benefit when treated. However, adherence represents a major challenge particularly in patients with mild OSA [15, 16]. The current study shows that daytime NMES is a viable option that is well accepted and associated with improvements in OSA severity and daytime sleepiness. Prior work on daytime NMES using observational and uncontrolled designs has demonstrated improvements in OSA severity, daytime sleepiness and snoring [8–11, 17]. The current randomised trial adds to the limited body of evidence by demonstrating that NMES can decrease OSA severity and daytime sleepiness. Clearly, studies over longer treatment periods based on larger samples are needed to characterise the heterogeneity of treatment response across the continuum of disease severity (*i.e.* from mild to severe OSA) and assess other health-related outcomes (*e.g.* blood pressure). An obvious advantage of NMES over PAP therapy is that the adherence is high given that it can be used during the day.

With any new therapy, there is the obvious need to assess its efficacy first. However, this study was designed to assess adherence to NMES as the primary outcome and not efficacy. Assessing adherence before determining efficacy is, in fact, valuable for several reasons. First, it is well established that adherence to PAP therapy is suboptimal, whether in a clinical trial or in the real world. Thus, understanding adherence patterns to a new form of therapy for OSA is of paramount significance before undertaking a large clinical trial to determine its efficacy. If the current study had revealed that patients with mild OSA do not use NMES, there would be no justification to conduct a randomised clinical trial given the associated burden on undertaking such an initiative. A second, and related, issue is that adherence itself is an important factor that mediates efficacy. For outcomes such as daytime sleepiness, there is little controversy that adherence dictates the degree of clinical improvement in patients with OSA. Thus, if adherence had been poor, it would have been challenging to address the issue of efficacy. Finally, it is crucial to acknowledge that this study was designed to also develop a sham device. A valid sham device has enormous value if it can mimic the intervention of interest without producing specific treatment effects. This study illustrates that a valid sham intervention for daytime NMES is indeed feasible. Differences in average stimulation setting between the sham and active group is not surprising given the lower voltage delivered even at the maximum stimulation intensity setting with the sham device. The availability of sham NMES with comparable adherence to the active NMES device will now allow for rigorous testing of its utility in other patient groups, including those with moderate or severe OSA.

The biological basis for daytime NMES improving OSA severity is not known. Skeletal muscles such as the quadriceps and hamstring demonstrate a transition from type II (fast-twitch) to type I (slow-twitch) muscle fibres after 2 months of low-frequency NMES [7]. In the New Zealand white rabbit, electrical stimulation of the genioglossus caused a phenotypic shift from fast- to slow-twitch fibres [18]. Given that endurance training induces a shift from type II to I muscle fibres, a transition to more slow-twitch fibres has value given that the tongue in patients with OSA has a greater proportion of fast-twitch fibres [19]. A larger proportion of type I fibres in the genioglossus is protective against fatigue and may decrease airway collapse during sleep. Whether the intensity and duration of electrical stimulation used in the current study leads to a transition from type II to I muscle fibres is unknown. A recent mechanistic study in mild OSA showed improvements in tongue endurance without any associated differences in average phasic genioglossus muscle activity during sleep [17]. Additional research on the effects of NMES on upper airway collapsibility in OSA is clearly needed.

There are several strengths in the current study. First, it was a parallel-group, double-masked and sham-controlled study. This approach minimises biases of selection, allocation and assessment. While there is a potential for limited generalisability, effects of confounding were minimised with a randomised design that includes a sham intervention with masking of participants and the research team. Second, the use of multi-night testing in mild OSA curtailed the bias from night-to-night variability in the assessment of disease severity [20–23]. Third, use of objective measures to assess improvements in the REI along with subjective sleepiness allows for clinical contextualisation of the observed changes. Limitations of this study include the small sample size, restriction to mild OSA and lack of follow-up beyond 6 weeks. A larger sample size with longer follow-up would help define the heterogeneity of treatment effects and assessment of the resiliency. These issues notwithstanding, the results of this study provide evidence on the adherence to active and sham NMES along with preliminary data on clinical efficacy to a novel treatment approach for mild OSA. Further work across the spectrum of OSA severity is needed to anchor the utility of daytime NMES in OSA.

## Supplementary material

10.1183/23120541.00474-2023.Supp1 **Please note:** supplementary material is not edited by the Editorial Office, and is uploaded as it has been supplied by the author.
Supplementary material 00474-2023.SUPPLEMENT
