# Supplementary material for: Neuromuscular electrical stimulation for obstructive sleep apnoea: comparing adherence to active and sham therapy
Source: ERJ Open Res. 2023 Dec 27;9(6):00474-2023. doi: 10.1183/23120541.00474-2023 (PMC10752290; doi:10.1183/23120541.00474-2023)
Supplement: Supplementary file 1 [file 00474-2023.SUPPLEMENT.pdf]

Supplementary Methods

Voltage Delivered for Various Settings for Active and Sham Neuromuscular Electrical Stimulation

The active NMES device employs a voltage driven system, with a maximum of 32.8 volts. The maximum of 6.6 volts was selected is associated with a mild sensation of a stimulus and was hypothesized to have no clinical effect. The following table describes the different intensity settings and the voltage delivered for each for the active and sham NMES device.

Setting	Active Voltage (V)	Sham Voltage (V)
1	2.19	0.44
2	4.37	0.88
3	6.56	1.32
4	8.75	1.76
5	10.93	2.20
6	13.13	2.64
7	15.31	3.08
8	17.49	3.52
9	19.68	3.96
10	21.87	4.40
11	24.05	4.84
12	26.24	5.28
13	28.43	5.72
14	30.31	6.11
15	32.80	6.60

Derivation of Sample Size

To determine total sample size, we assumed that the proportion of patients using the device as recommended (42 days) in the study would be approximately 0.85. Additionally, we wanted to estimate that proportion with a precision of approximately 10-12%. Thus, a margin of error (MOE) was assumed at ± 0.12. The sample size for a proportion is then determined by:

$$n = \frac{Z^2}{MOE^2}(\tilde{p} * (1 - \tilde{p}))$$

where $\tilde{p}$ = 0.85, Z = 1.96, and MOE = 0.12. The resulting total sample size would be 34 with equal number in the sham and active NMES group (N=17 per group). To account for potential

dropout, the sample size was augmented by 15% yielding a target recruitment of 40 patients for the study.

Statistical Analysis

In addition to the use of mixed-effects linear regression, which was employed to model changes in the respiratory event index (REI) with neuromuscular electrical stimulation (NMES), a Bayesian approach to the mixed model was also used as part of sensitivity analyses for modeling the change in daytime sleepiness (i.e., Epworth Sleepiness Scale [ESS] score) with NMES. The limitations of a small sample size of the current study, which likely led to the marginal statistical significance observed in improvements of daytime sleepiness (i.e., Epworth Sleepiness Scale [ESS] score), can be more rigorously explored with a Bayesian approach. A mixed model contextualized with a Bayesian approach does not require any assumptions about the underlying distribution of the outcome variable (i.e., ESS). With this method, the posterior distribution of the parameter relating NMES to changes in the ESS score is estimated using non-informative priors. The specification of the Bayesian model was identical to the mixed-effects model which included an indicator variable for the interaction between the assigned arm (active vs. sham NMES) and visit (baseline vs. 6-weeks) along with a covariate for body mass index, which proved to be the only significant predictor of change in ESS scores at 6-weeks. An indicator variable was also included for the patient as a random effect. In contrast to the mixed-effects linear regression, the Bayesian mixed model approach relies on simulations to estimate the posterior distribution of parameters. Therefore, summary statistics from this approach are not reported as a point estimate along with a 95% confidence interval, but rather a point estimate with a 95% credibility interval.

Supplementary Results

Table S1. Change in the ESS score (95% credibility interval) in Bayesian mixed-effects linear regression model with ESS as the outcome

	Active NMES Arm	Sham NMES Arm	p-value
Change (Δ) in ESS	-1.8 (-2.8, -0.8)	-0.9 (-1.9, 0.2)	0.04

NMES: Neuromuscular electrical stimulation. P-values are derived for comparisons of the Δ_{Active} to Δ_{Sham} from the mixed linear regression model.

Longitudinal Follow-up

The overarching goal of the study was to determine adherence to active and sham NMES at 6 weeks. Thus the primary endpoint was the proportion of patients who are adherent at the completion of the study. The protocol did not include any recommendations or plans for additional longitudinal follow-up at the end of 6 weeks. Nonetheless, we queried the device manufacturer’s database and noted that 11 participants from the active and 7 participants from the sham arm decided to pursue therapy on their own. The average number of days therapy was used by the 11 and 7 participants was 89 days and 101 days, respectively.
